# Association of Acute Symptoms of COVID-19 and Symptoms of Depression in Adults

**DOI:** 10.1001/jamanetworkopen.2021.3223

**Published:** 2021-03-12

**Authors:** Roy H. Perlis, Katherine Ognyanova, Mauricio Santillana, Matthew A. Baum, David Lazer, James Druckman, John Della Volpe

**Affiliations:** 1 Massachusetts General Hospital, Boston; 2 Harvard Medical School, Boston, Massachusetts; 3 Associate Editor, *JAMA Network Open*; 4 Rutgers University, New Brunswick, New Jersey; 5 Harvard University, Cambridge, Massachusetts; 6 Northeastern University, Boston, Massachusetts; 7 Northwestern University, Evanston, Illinois

## Abstract

This survey study investigates where acute coronavirus disease 2019 (COVID-19) is associated with the probability of subsequent depressive symptoms among US adults.

## Introduction

After acute infection with severe acute respiratory syndrome coronavirus 2 (SARS-CoV-2), a subset of individuals experience persistent symptoms involving mood, sleep, anxiety, and fatigue,^1^ which may contribute to markedly elevated rates of major depressive disorder observed in recent epidemiologic studies.^2^ In this study, we investigated whether acute coronavirus disease 2019 (COVID-19) symptoms are associated with the probability of subsequent depressive symptoms.

## Methods

In this survey study, we included data from US adult participants in 8 waves of an internet-based nonprobability survey conducted by Qualtrics with multiple panels of respondents (PureSpectrum). Surveys were conducted approximately monthly between June 2020 and January 2021. Of 82 319 respondents who completed the Patient Health Questionnaire–9 (PHQ-9), a total of 3904 nonoverlapping individuals reported prior COVID-19 illness and completed the survey questions used in this analysis. The study was reviewed and approved by the institutional review board of Harvard University. All participants signed electronic informed consent. We followed the American Association for Public Opinion Research (AAPOR) reporting guideline for survey studies.

In addition to standard sociodemographic questions, including self-identified race and ethnicity in 5 prespecified categories based on the US Census, the survey asked participants whether they had been diagnosed with COVID-19 illness by a clinician or received a positive test result and in which month(s) they had been ill; these individuals were also asked to indicate the presence or absence of specific symptoms and overall perceived severity of COVID-19 illness (ie, not at all, not too, somewhat, or very). Participants also completed the PHQ-9, a screen for symptoms of depression,^3^ with each of the 9 items scored from 0 to 3, yielding a score between 0 and 27; a score of 10 or greater is considered moderate depression.

For the primary analysis, we incorporated indicator variables for each symptom as well as overall severity in a logistic regression model with PHQ-9 score of 10 or greater (ie, moderate or greater depression) as the dependent variable. We then adjusted for sociodemographic features including age, gender, race/ethnicity, geographic region, urban vs suburban or rural, and household income, using glm package in R version 3.6 (R Project for Statistical Computing). Statistical significance was set at α = .05, and all tests were 2-tailed.

## Results

There were 3904 individuals reporting prior COVID-19 illness (Table), with a mean (SD) age of 38.1 (12.4) years. Overall, 1730 (44.3%) were women; 416 (10.7%), Hispanic individuals; 439 (11.2%), Black individuals; and 142 (3.6%), Asian individuals. Mean (SD) time since initial COVID-19 symptoms was 4.2 (2.7) months. A total of 2046 participants (52.4%) met the criteria for symptoms of major depressive disorder. In fully adjusted models, presence of headache was associated with greater probability of moderate or greater depression symptoms (adjusted odds ratio [OR], 1.33; 95% CI, 1.10-1.62), as was greater overall severity (somewhat vs not at all severe: adjusted OR, 2.59; 95% CI, 2.04-3.30; very vs not at all severe: OR, 5.08; 95% CI, 3.93-6.59). Women were less likely to have symptoms than men (adjusted OR, 0.72; 95% CI, 0.61-0.84), and the likelihood of symptoms decreased with increasing age (adjusted OR by decade, 0.76; 95% CI, 0.72-0.81). The Figure illustrates ORs from regression models adjusted for sociodemographic features, omitting 288 individuals who lacked overall COVID-19 severity data.

**Table.** Demographic Information and Acute COVID-19 Symptoms Stratified by PHQ-9 Depression Score

Characteristic	Patients by PHQ-9 Score, No. (%)		Total (n = 3904), No. (%)	*P* value
	<10 (n = 1858)	≥10 (n = 2046)		
Positive test^a^	1453 (78.9)	1399 (69.4)	2852 (74.0)	<.001
Clinician diagnosis^b^	1586 (85.5)	1758 (86.0)	3344 (85.7)	.62
Time since onset, mean (SD), mo^c^	3.75 (2.61)	4.56 (2.80)	4.18 (2.74)	<.001
Age, mean (SD), y	39.92 (13.59)	36.36 (10.92)	38.05 (12.39)	<.001
Female gender	927 (49.9)	803 (39.2)	1730 (44.3)	<.001
Income median (IQR), thousands of $^d^	70 (30-125)	75 (30-150)	75 (30-145)	.002
Race/ethnicity				
White	1320 (71.0)	1468 (71.7)	2788 (71.4)	.20
Hispanic	195 (10.5)	221 (10.8)	416 (10.7)	
Black	203 (10.9)	236 (11.5)	439 (11.2)	
Asian	71 (3.8)	71 (3.5)	142 (3.6)	
Other	69 (3.7)	50 (2.4)	119 (3.0)	
Urbanicity				
Rural	275 (14.8)	264 (12.9)	539 (13.8)	.02
Suburban	1009 (54.3)	1071 (52.3)	2080 (53.3)	
Urban	574 (30.9)	711 (34.8)	1285 (32.9)	
Acute COVID-19 severity^e^				
Not at all severe	317 (18.7)	153 (7.9)	470 (13.0)	<.001
Not too severe	553 (32.7)	398 (20.6)	951 (26.3)	
Somewhat severe	558 (33.0)	709 (36.8)	1267 (35.0)	
Very severe	263 (15.6)	669 (34.7)	932 (25.7)	
Symptoms				
Fever	946 (50.9)	1083 (52.9)	2029 (52.0)	.21
Chills	641 (34.5)	758 (37.0)	1399 (35.8)	.10
Shaking	590 (31.8)	714 (34.9)	1304 (33.4)	.04
Congestion	948 (51.0)	1051 (51.4)	1999 (51.2)	.83
Muscle pain	636 (34.2)	786 (38.4)	1422 (36.4)	.007
Cough	749 (40.3)	799 (39.1)	1548 (39.7)	.42
Sore throat	813 (43.8)	925 (45.2)	1738 (44.5)	.36
Headache	403 (21.7)	603 (29.5)	1006 (25.8)	<.001
Shortness of breath	673 (36.2)	842 (41.2)	1515 (38.8)	.002
Change in taste or smell	980 (52.7)	1122 (54.8)	2102 (53.8)	.19

Abbreviations: COVID-19, coronavirus disease 2019; PHQ-9, Patient Health Questionnaire–9.

^a^ Missing in 17 participants (0.9%) with PHQ-9 scores of less than 10 and 31 participants (1.5%) with PHQ-9 scores of 10 or greater.

^b^ Missing in 2 participants (0.1%) with PHQ-9 scores of less than 10 and 2 participants (<0.1%) with PHQ-9 scores of 10 or greater.

^c^ Missing in 249 participants (13.4%) with PHQ-9 scores of less than 10 and 213 participants (10.4%) with PHQ-9 scores of 10 or greater.

^d^ Missing in 1 participant (<0.1%) with PHQ-9 scores of less than 10 and 3 participants (0.1%) with PHQ-9 scores of 10 or greater.

^e^ Not asked in 167 participants (9.0%) with PHQ-9 scores of less than 10 and 117 participants (5.7%) with PHQ-9 scores of 10 or greater.

**Figure.**
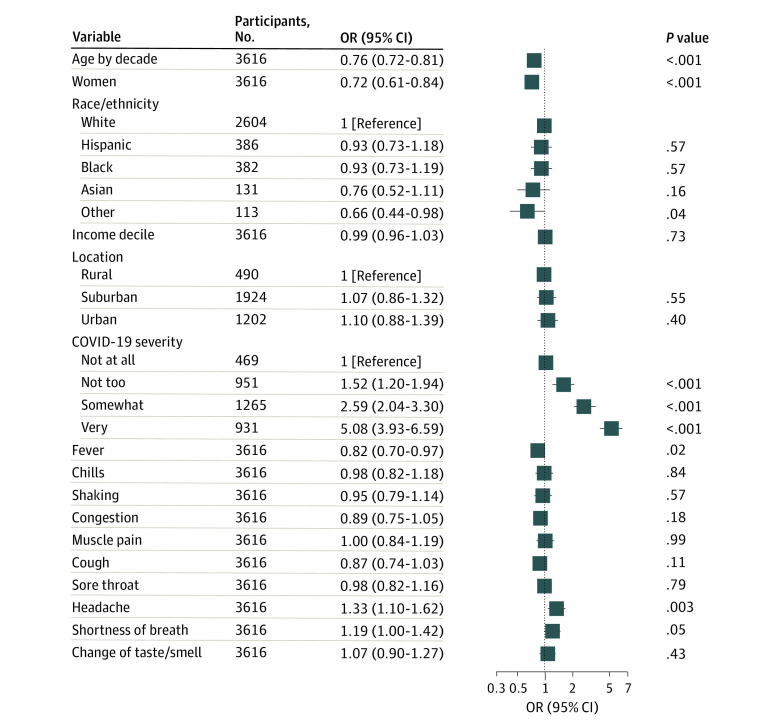
Logistic Regression Model for Association of Acute Coronavirus Disease 2019 (COVID-19) Symptoms With Subsequent Patient Health Questionnaire–9 Depression Score OR indicates odds ratio.

## Discussion

Among more than 3900 individuals with prior COVID-19 illness surveyed between May 2020 and January 2021, 52.4% met criteria for moderate or greater symptoms of major depression. In regression models, these symptoms were more likely among younger respondents compared with older respondents and among men compared with women as well as among those with greater self-reported overall COVID-19 severity compared with those with lower severity.

We did not replicate a prior finding^4^ among 114 individuals with COVID-19 that loss of smell and taste were associated with greater near-term depressive and anxious symptoms. Instead, we found that those who reported headache during acute infection appeared to have an elevated risk of depressive symptoms. We note the important caveat that, as a cross-sectional study, we cannot exclude the possibility that individuals with current depression are more likely to recall or report headache. We might similarly expect other symptoms to also be reported more frequently, but this was not generally the case. Moreover, as a web-based survey, we cannot estimate a response rate as with more traditional survey designs; however, we note that surveys using similar methods have demonstrated replicable results during COVID-19.^5^ As respondents did not see the survey topic until entering the survey itself, it is unlikely our results are enriched for those with particular interest in, or impact from, COVID-19.

A further caveat is that we cannot attribute these symptoms to new onset of depression; individuals with acute infection could be less likely to recover from prior depressive episodes or those with preexisting depressive symptoms could have greater risk of contracting COVID-19. A 2021 claims-based study^6^ suggests a bidirectional association between COVID-19 and psychiatric illness.

Nevertheless, our results add to a growing body of evidence suggesting the importance of considering potential neuropsychiatric sequelae of COVID-19 infection. Our results also suggest the importance of considering strategies that might mitigate the elevated risk of depressive symptoms following acute infection.
